# Prophylactic treatment with *Bacteroides uniformis* and *Bifidobacterium bifidum* counteracts hepatic NK cell immune tolerance in nonalcoholic steatohepatitis induced by high fat diet

**DOI:** 10.1080/19490976.2024.2302065

**Published:** 2024-01-09

**Authors:** Jingyuan Xu, Qiaoyun Xia, Ting Wu, Yong Shao, Yatao Wang, Nuyun Jin, Peiying Tian, Longyun Wu, Xiaolan Lu

**Affiliations:** a Department of Gastroenterology, Shanghai Pudong Hospital, Fudan University Pudong Medical Center, Shanghai, China; b Department of Gastroenterology, the Affiliated Suzhou Hospital of Nanjing Medical University, Suzhou, China; c Department of Gastroenterology, The Second Affiliated Hospital of Xi’an Jiaotong University, Xi’an, China; d Department of Citizen Health, Community Health Service Center of Jinxi Town, Kunshan, China

**Keywords:** *Bacteroides uniformis*, *Bifidobacterium bifidum*, natural killer cell, immune tolerance, nonalcoholic steatohepatitis

## Abstract

Hepatic immunity is one of the driving forces for the development of nonalcoholic steatohepatitis (NASH), and targeting gut microbiota is believed to affect the hepatic immune constitution. Here, we aimed to investigate the hepatic immunological state in NASH, with a specific emphasis on natural killer (NK) cells. In addition, we aimed to identify the contributing species that target hepatic immunity to provide new directions and support the feasibility of immunotherapy for NASH. A possible NASH population was determined by combination of long-term severe fatty liver, metabolic disorders and increased serum CK18 to detect serum immune factors and gut microbiota. NASH was induced in mice fed a high-fat diet to verify the prophylactic effect of the functional species on the immunopathology and development of NASH. Hepatic immunologic state was examined, and the effector functions of NK cells were detected. Hepatic transcriptome, proteomic, and fecal metagenome were performed. We observed a statistical increase in serum IL-10 (p < 0.001) and non-statistical decrease in interferon-γ and IL-6 in NASH population, hinting at the possibility of immune tolerance. Fecal Bacteroides uniformis and Bifidobacterium bifidum were abundant in healthy population but depleted in NASH patients. In NASH mice, hepatic CD8+T cells, macrophages, and dendritic cells were increased (p < 0.01), and NK cells were inhibited, which were identified with decreased granzyme B (p < 0.05). Bacteroides uniformis and Bifidobacterium bifidum improved hepatic pathological and metabolic cues, increased hepatic NK cells and reduced macrophages (p < 0.05). Bacteroides uniformis also restored hepatic NK cell function, which was identified as increased CD107a (p < 0.05). Transcriptional and translational profiling revealed that the functional species might restore the function of hepatic NK cells through multiple pathways, such as reduction of inhibitory molecules in NK cells. Bacteroides uniformis and Bifidobacterium bifidum are novel prophylactics for NASH that restore the impaired function of hepatic NK cells.

## Introduction

Humanity is faced with a stark increase in the incidence of nonalcoholic fatty liver disease (NAFLD), representing a spectrum of disorders ranging from simple steatosis to hepatocellular carcinoma (HCC) over time.^1,2^ Nonalcoholic steatohepatitis (NASH), characterized by hepatocyte steatosis, inflammation, and ballooning, is a pivotal stage of NAFLD and may be the most common cause of the development of liver cirrhosis and HCC in the future.^3,4^ Currently, the pathogenesis of NASH is unclear, and no drug has been approved for clinical application. A better understanding of the mechanisms underlying NASH is essential for developing therapeutic strategies. It is known that the dysregulation of different innate and adaptive immune cell compartments plays an indispensable role in the pathogenesis of NASH, such as natural killer (NK) cells.^5^

NK cells, which kill pathogens, drive antitumor immune responses and stress hepatocytes via innate immune recognition. Cytokines such as interferon-γ (IFN-γ), tumor necrosis factor (TNF), chemokines, and growth factors are critical components of the liver innate immune system.^5^ Recently, increasing attention has been given to the role of NK cells in NASH. Some researchers have found that IFN-γ derived from NK cells is critical for the maintenance of a balanced inflammatory environment, which preserves liver tissue integrity and retards fibrosis progression.^6^ Consistently, a clinical study showed that the frequency of circulating NK cells in obese people was lower than that in non-obese people,^7^ accompanied by NK cell dysfunction, especially a decline in cytotoxic activity.^5^ In contrast to these findings, animal studies have shown that hepatic NK cells are excessively activated in NASH mice induced by a methionine and choline-deficient diet (MCD), choline-deficient high-fat diet (CDHFD), and high-fat diet (HFD) with streptozotocin injection, thereby exacerbating hepatocellular damage.^8^ However, the function of hepatic NK cells in an HFD-induced NASH mouse model has not yet been reported. Therefore, it is necessary to further clarify the role of NK cells and reveal their possible mechanisms in an HDF-induced NASH model, which is closer to the pathogenesis of patients with NASH. Currently, researchers have confirmed that the function of NK cells may be compromised by specific circumstances and factors such as the tumor microenvironment and TGF-β. The tumor microenvironment can induce the expression of inhibitory receptors and downregulate activating receptors involved in tumor cell recognition and killing, leading to impaired antitumor activity of NK cells.^9,10^ TGF-β converts effector NK cells into type 1 innate lymphocytes, leading to tumor immune evasion.^11^

There is a growing appreciation that the gut microbiota has a crucial impact on the function of NK cells.^12,13^ Studies have shown that early-life antibiotic treatment can blunt the functional maturation of liver-resident NK cells even in adulthood.^13^ Postbiotics increase NK cell activation and exert potent protection in the maintenance of the intestinal microecological system.^14^ Moreover, a meta-analysis of randomized controlled trials found that probiotics substantially invigorated NK cells in the elderly.^15^ Although existing studies have suggested the potential of the gut microbiota to modulate NK cell immunological function, practical studies are scarce.

To clarify the role of NK cells in NASH and determine whether the gut microbiota can affect NASH by regulating NK cells, we identified potential probiotics from volunteers and verified their effects on NASH and NK cell immune function by gavage in high-fat diet (HFD)-induced NASH mice. In addition, the fecal metagenome, hepatic transcriptome, and proteome were explored to discover the relevant mechanisms regarding both NK cell activation and alleviation of NASH, hoping to provide a reference for the clinical treatment of NASH.

## Methods

### Study populations

Based on the premise of passing the review of the Ethics Committee of the Biomedical Department of Fudan University Pudong Medical Center, we recruited 20 NASH patients according to the following criteria: (1) age between 25 and 65 years; (2) history of severe fatty liver for over 10 years; and (3) at least two types of metabolic disorders, including overweight/obesity, hyperglycemia, hypertension, hyperlipidemia, and hyperuricemia. The exclusion criteria were as follows: (1) pregnancy; (2) diagnosis of other causes of chronic liver disease, including viral hepatitis, autoimmune liver disease, and alcoholic fatty liver disease (>20 g/day or women, >30 g/day for men); (3) antibiotic use in the past 6 months; and (4) terminal or other malignant diseases. At the same time, we recruited 20 healthy subjects aged 25–65 from the same communities as the healthy group (matched pair). All the participants provided written informed consent.

### Plasma cytokine measurements

Blood samples were collected and centrifuged at 3000 rpm for 15 min before collecting the supernatant. The CK-18 kit (MM-1447H1) was purchased from Jiangsu Meimian Industrial Co. Ltd. ELISA kits for IFN-γ (EK106HS–01), IL-10 (EK110HS–01), and IL-6 (EK180HS–01) were purchased from Hangzhou Lianke Biotechnology Co., Ltd. The levels of the relevant indicators were determined according to the manufacturer’s instructions.

### Feces collection

After obtaining the consent of each research subject, we provided a sterilized disposable bedpan and waited since 4 a.m. After the subject woke up and defecated in the morning, we used a high-temperature and high-pressure sterilized stainless steel spoon to remove the upper layer of feces immediately. Feces were collected in a sterile frozen storage tube and immediately frozen in liquid nitrogen. All samples were collected within one week and were stored at −80°C before testing.

Mouse feces were collected on an ultraclean table. Each mouse was placed on sterile gauze alone, and the feces were immediately collected into frozen tubes with sterile forceps for quick freezing. Samples were stored at −80°C before testing.

### Fecal 16S rRNA sequencing

Total genomic DNA of the microbial community was extracted using a Mag Bind DNA Kit (M9636–02, Omega Bio Tek). After qualification and purification, PCR amplification was performed on the V3-V4 variable region, and 2% agarose gel was used to recover products. Subsequently, the recovered products were purified and quantified, and a NEXTFLEX Rapid DNA-Seq Kit was used to build a library of purified PCR products. Fastp (https://github.com/OpenGene/fastp) was used for quality control, FLASH (http://www.cbcb.umd.edu/software/flash) was used for splicing, and operational taxonomic unit (OTU) clustering was performed on the quality control spliced sequences based on 97% similarity. Data was deposited into the NCBI Sequence Read Archive (SRA) database (Accession Number: PRJNA1034345).

### Bacterial culture

*Bacteroides uniformis* (*B. uniformis*) (BNCC139204) and *Bifidobacterium bifidum* (*B. bifidum*) (BNCC186304) were purchased from BeNa Culture Collection and cultured anaerobically (Mitsubishi anaeropack and anaerobic culture bag) with Columbia blood plates and BBL liquid culture medium at 37°C, respectively.

### Animals

All animal experiments were performed in accordance with protocols approved by the Animal Experimentation Ethics Committee of Fudan University Pudong Medical Center. Male wild-type C57BL/6 mice were obtained at four weeks and randomly divided into two groups: healthy and NASH-model. The animals were maintained in an SPF animal room at a constant temperature and humidity, allowing free access to food and water. The healthy group was fed a regular chow diet, whereas the NASH-model group was fed a 60% HFD (Changzhou SYSE Bio-Tech. Co., LTD.) for 45 weeks to establish the NASH model. On this basis, 30 four-week-old C57BL–6 mice were purchased and randomly divided into five groups: normal chow diet (NCD), Health-FMT, NASH-FMT, Bifi, and Uni groups, with six mice in each group. Mice in the NCD group were fed an NCD, while the others were fed an HFD. An antibiotic cocktail (ABX) was administered to the mice by gavage for 2 weeks to eliminate the intestinal microbiota and construct a pseudo-sterile mouse model. The composition of ABX was as follows: vancomycin (Sigma-Aldrich,1404-93-9) 200 mg/kg, gentamicin (Sigma-Aldrich, 1405-41-0) 25 mg/kg, penicillin (Sigma-Aldrich, V900929) 625 mg/kg, and amphotericin B (Sigma-Aldrich,1397-89-3) 7.5 mg/kg. The Health-FMT and NASH-FMT groups were transplanted with the whole fecal bacteria of the healthy and NASH-model groups, respectively. The Bifi and Uni groups were gavaged with 1 × 10^8^ CFU of *B. uniformis and B. bifidum*, respectively. Each mouse was administered omeprazole (Sigma-Aldrich 73,590-58-6) 10 mg/kg intraperitoneal injection half an hour before gavage to reduce the lethality of gastric acid on the bacteria. All mice were administered once every other day for eight weeks and continuously fed the current diet for 52 weeks. At the end of the experiment, mice were fasted (12 hours), weighed, and sacrificed using excessive pentobarbital. Blood, liver, colon, and white adipose tissue of the epididymis (eWAT) were collected and placed at − 80°C for subsequent experiments. The body length, colon length, body weight, eWAT weight, and liver weight were measured. Lee’s index was calculated as (weight (g) × 1000) ^ (1/3)/body length (cm). The liver index was calculated as liver weight/body weight.

To further explore whether *B. uniformis and B. bifidum* can restore hepatic NK cell inhibition by long-term HFD, 5-week-old C57BL/6 mice were fed a NCD (Normal group, *n* = 6) or HFD for 46 weeks. Subsequently, mice fed a HFD were randomly divided into three groups: NASH group (*n* = 5, gavaged with 0.2 ml PBS once every other day), Uni-treat group (*n* = 5, gavaged with 1 × 10^8^ CFU of *B. uniformis* once every other day), and Bifi-treat group (*n* = 5, gavaged with 1 × 10^8^ CFU of *B. bifidum* once every other day). After another 8 weeks, the mice were sacrificed and the liver tissues and feces were collected as described above.

### Biochemical index detection

Alanine transaminase (ALT) (Leidu, S03030), aspartate transaminase (AST) (Leidu, S03040), γ-glutamyl transpeptidase (γ-GT) (Leidu, S03031), fasting blood glucose (FBS) (Leidu, S03039), triglycerides (TG) (Leidu, S03027), low-density lipoprotein cholesterol (LDL) (Leidu, S03029), high-density lipoprotein cholesterol (HDL) (Leidu, S03025), and glycated serum protein (GSP) (Leidu, C024) levels were detected using an automatic biochemistry analyzer (Leidu, Chemray 800).

### Histology and immunochemistry

The liver tissue was fixed with 4% paraformaldehyde, embedded in paraffin, and then sectioned into 4 μM thick slices. Hematoxylin and eosin (H&E) staining, reticular fiber staining, and Masson staining were performed according to the standard dyeing scheme. Immunohistochemistry was used to measure the levels of hepatic CK18 (Servicebio, GB11232), colorectal Zonula occludens protein 1 (ZO-1) (Abcam, ab190085), and occludin (Abcam, ab216327).

### Multi-color flow cytometry analysis

Fresh livers were ground with a 70 mm cell strainer to obtain a uniform cell suspension. The cell suspension was centrifuged at 350 g for 5 min before the supernatant was discarded. The cell precipitate was resuspended in 40% Percoll, gently layered on a gradient of 70% Percoll, and centrifuged at 1260 g for 30 min. Finally, the cell layers were aspirated, resuspended, and counted.

For the staining of surface proteins, the cells isolated from the livers of mice were adjusted to a concentration of 1 × 10^7^ cells/mL and vortexed after dyeing. Subsequently, the cells were incubated in the dark for 15 min and centrifuged at 350 g for 5 min before discarding the supernatant. After resuspension, the blocking agent and corresponding antibodies were added (Supplementary Table S1). After surface staining, a fixation buffer was added. The cells were incubated at room temperature in the dark for 20 min before centrifugation, and the supernatant was discarded. Subsequently, the cells were resuspended in Perm buffer, centrifuged, and resuspended in Perm wash. The corresponding antibodies (Supplementary Table S1) were added, and the cells were incubated in the dark. Data were acquired and analyzed using CytoFLEX software.

### Transcriptome analysis

RNA in liver tissue was isolated and purified by TRIzol (Invitrogen, CA). After the quality control of total RNA quantity and integrity, the mRNA containing PolyA was specifically captured through two rounds of purification using Oligo (dT) magnetic beads (25–61005, Thermo Fisher, USA). The mRNA was captured under high-temperature conditions for fragmentation. The fragmented RNA was transcribed and U-labeled second-stranded DNAs was synthesized, and digested with UDG enzyme (NEB, m0280, MA, US). PCR was performed to form a library with a fragment size of 300 bp ±50 bp and an Illumina Novaseq ™ 6000 (LC Bio-Technology Co., Ltd. Hangzhou, China) was employed to perform double-ended sequencing according to standard operations, with the sequencing mode being PE150. Fastp was used to perform quality control. HISAT2 was used to compare sequencing data to the genome. StringTie was used to assemble genes or transcripts and quantify them using FPKM. The differentially expressed mRNAs were selected with fold change > 2 or fold change < 0.5 and with parametric F test comparing nested linear models (*p* < 0.05) by R package edgeR. Data was deposited into the NCBI Sequence Read Archive (SRA) database (Accession Number: PRJNA1045022).

### Metagenomic sequencing

DNA was extracted by a Mag Bind DNA Kit (Omega Bio tek, USA) and qualified by 1% Agarose gel electrophoresis. Extracted DNA was fragmented by Covaris M220 (Gene Company Limited, China) and screened at approximately 400 bp fragments for paired-end library construction. NEXTFLEX Rapid DNA-Seq (Bioo Scientific, USA) was used to build paired-end library construction. The Illumina NovaSeq (Illumina, USA) sequencing platform was used for metagenomic sequencing. Fastp was used to perform quality cropping on the adapter sequences of the 3‘and 5’ ends of reads, preserving high-quality paired-end reads and single-end reads. BWA (http://bio-bwa.sourceforge.net) was used to construct the reads with the host DNA sequence and remove contaminated reads with high alignment similarity. MEGAHIT (https://github.com/voutcn/megahit) was used to splice and assemble the optimized sequence. SOAP aligner (http://soap.genomics.org.cn/) was used to compare the high-quality reads of each sample with the non-redundant gene set (95% identity) and calculate the abundance information of genes in the corresponding samples. Diamond (http://www.diamondsearch.org/index.php) was used to annotate species taxonomy, COG function, and KEGG function. Data was deposited into the NCBI Sequence Read Archive (SRA) database (Accession Number: PRJNA1035813).

### Proteomics

The tissue was cut and an appropriate amount of SDT lysate was added. An MP FastPrep-24 Automated Homogenizer was used to crush and homogenize liver tissue (24 × 2, 6.0 M/s, 60 s, twice). After boiling (10 minutes), the tissue homogenate was centrifuged at 14,000 × g for 15 minutes. Subsequently, the supernatant was taken and filtered using a 0.22 µm centrifuge tube to collect the filtrate. After quantification and enzymatic hydrolysis, the peptide segment was desalinated using a C_18_ cartridge. The peptide mixture of all samples was taken and subjected to high-PH RP grading using the Agilent 1260 infinity II HPLC system. Components were collected, and each was dried in a vacuum concentrator. After freeze-drying, the sample was redissolved with 0.1% formic acid aqueous solution and combined into six fractions. After tandem mass spectrometry analysis, the original mass spectrometry data were merged and analyzed using Spectronaut Pulsar X (version 12, Biognosys AG) to establish a spectrogram database.

### Agarose gel electrophoresis

Fecal DNA was extracted by a TIAAmp Atool DNA Kit (TIANGEN, DP328) according to the manufacturer’s manual. After detecting the concentration, 450 ng of DNA was added to the octopus tube, and 25 µl of 2×Pro Taq master mix was added, with 1ul of forward primer (*B. uniformis*: F-AGTAGAGGCAGGCGGAAT; *B. bifidum*: F-TGAAAGTCCATCGCTTAA) and backward primer (*B. uniformis*: R-CGAGCATCAGCGTCAGTT; *B. bifidum*: R-CTACACATTCCACCGTTA), respectively, and ddH2O was added to supplement the mixture to 50 µl. After reverse transcription was completed, 6×lodding buffer was added and mixed well for later use. The samples were added to 2% agarose gel under 120 V constant pressure and displayed in the gel imager after electrophoresis.

### Statistical analysis

Statistical analyses were conducted using GraphPad Prism 8.0 Software (GraphPad, La Jolla, CA, USA). Data with a normal distribution and skewed distribution were expressed as the mean ± standard deviation and median ± interquartile range, respectively. Comparisons between two groups were analyzed using the two-tailed Student’s t test and Mann-Whitney U test, according to the data distribution. Comparisons among groups were analyzed using a one-way ANOVA test, and least significant difference (LSD) was used in the post hoc test. Differences were considered statistically significant at *p* < 0.05.

## Results

### Immune tolerance may exist in NASH patients, with significant depletion of *B. uniformis* and *B. bifidum* in feces

**Figure 1.**
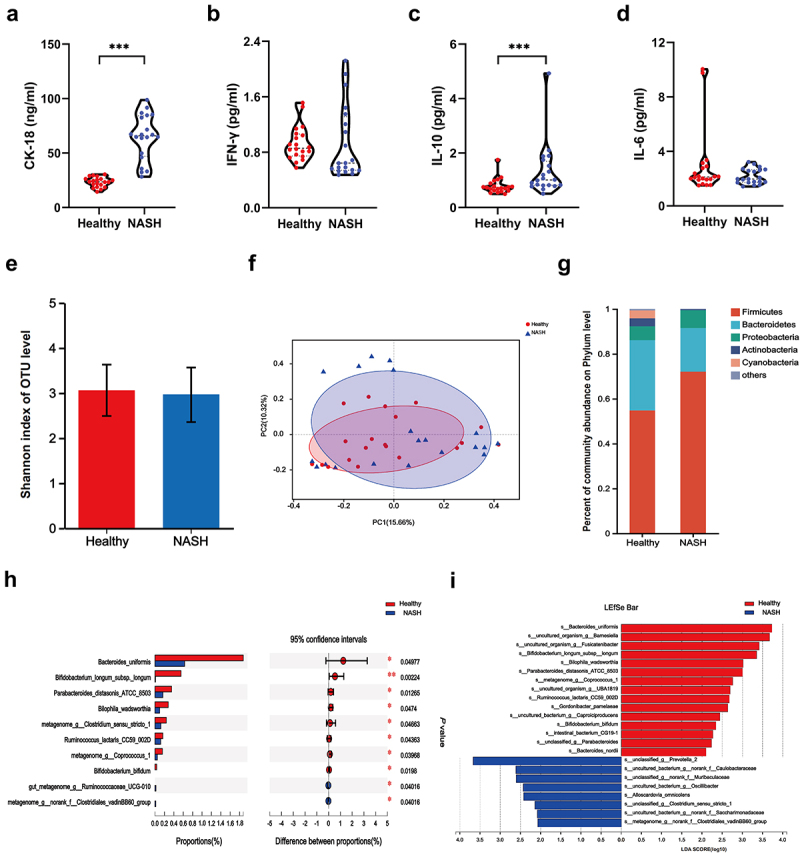
Immune tolerance and gut microbiota alteration in NASH group. (a) Serum CK-18 detected by ELISA. Compared with the healthy group, *p < 0.05, **p < .01, ***p < .001. (b) Serum IFN-γ detected by ELISA. Compared with the healthy group, *p < .05, **p < .01, ***p < .001. (c) Serum IL-10 detected by ELISA. Compared with the healthy group, *p < .05, **p < .01, ***p < .001. (d) Serum IL-6 detected by ELISA. Compared with the healthy group, *p < .05, **p < .01, ***p < .001. (e) Shannon Index at the OTU level. (f) Principal coordinates analysis of healthy group and NASH group. The X axis and Y axis represent the two selected principal axes, and the percentage represents the interpretation value of the principal axes for the difference in sample composition; the scale of the X axis and Y axis is relative distance and has no practical significance; points of different colors or shapes represent samples of different groups. The closer the two sample points are, the more similar the species composition of the two samples is. (g) Community bar plot analysis at the phylum level. The abscissa is the sample name, and the ordinate is the proportion of the phylum in the sample. The columns of different colors represent different phylum, and the length of the columns represents the proportion of the phylum. (h) Wilcoxon rank-sum test bar plot at the species level. The boxes of different colors represent different groups, and the length represents the average relative abundance of a species in different groups. (i) LDA discrimination column chart at the species level. The LDA discrimination column chart counts the microbial groups with significant effects in different groups. The greater the LDA score is, the greater the impact of the representative species abundance on the difference effect.

Metabolic disorders hasten the progression of simple fatty liver to NASH.^16,17^ Patients with more than 10 years of severe NAFLD and at least two types of metabolic disorders, including overweight/obesity, hyperglycemia, hypertension, hyperlipidemia, and hyperuricemia, were assigned to the NASH group (Table 1). To exclude the impact of diet and geography on the composition of gut microbiota, we screened healthy subjects in the same community as the healthy group (the relevant information of the subjects is shown in Tables 1 and 2). As expected, serum CK-18 levels markedly increased in the NASH group (Figure 1a). However, compared to the healthy group, serum IFN-γ and IL-6 in the NASH group showed no statistical significance, and IL-10 was statistically higher, hinting at an immunological tolerance in the NASH group (Figure 1b–d).

**Table 1.** Metabolic disorders in participants.

Personnel number	gender	age	Dyslipidemia	Overweight/Obesity	Hypertension	Hyperglycemia	hyperuricemia
S1	female	52	TG 2.53 mmol/L	BMI 29.97 Kg/㎡	93/141 mmHg	NO	NO
S2	female	57	TG 3.76 mmol/L	BMI 27.89 Kg/㎡; waistline 86 cm	95/185 mmHg	NO	NO
S3	male	56	TC 5.88 mmol/L; TG 2.28 mmol/L; LDL 3.74 mmol/L	BMI 26.26 Kg/㎡	NO	NO	NO
S4	female	64	TG 3.26 mmol/L; LDL 3.26 mmol/L	BMI 28.07 Kg/㎡	96/158 mmHg	HbA1c 6.5%	NO
S5	male	55	TG 2.20 mmol/L	BMI 29.03 Kg/㎡; waistline 98 cm	93/150 mmHg	HbA1c 8.5%; FBG 9.62 mmol/L	NO
S6	male	52	TG 2.85 mmol/L; LDL 3.27 mmol/L	BMI 24.51 Kg/㎡;	104/142 mmHg	NO	570.5 μmol/L
S7	female	62	TC 5.99 mmol/L; TG 3.00 mmol/L; LDL 3.71 mmol/L	BMI 25.39 Kg/㎡	84/175 mmHg	NO	NO
S8	female	60	TG 1.97 mmol/L	BMI 32.44 Kg/㎡; waistline 94 cm	88/169 mmHg	NO	380.3 μmol/L
S9	male	53	TG 2.73 mmol/L	BMI 26.89 Kg/㎡	NO	HbA1c 6.8%; FBG 6.39 mmol/L	NO
S10	male	60	TG 2.25 mmol/L; LDL 3.14 mmol/L	BMI 26.37 Kg/㎡; waistline 91 cm	NO	NO	NO
S11	male	54	TC 5.89 mmol/L; TG 6.62 mmol/L	BMI 25.71 Kg/㎡	NO	NO	NO
S12	male	49	TG 6.29 mmol/L	BMI 25.10 Kg/㎡; waistline 100 cm	102/163 mmHg	FBG 6.13 mmol/L	NO
S13	female	55	TG 3.84 mmol/L	BMI 24.84 Kg/㎡	91/153 mmHg	NO	NO
S14	male	51	TC 6.60 mmol/L; TG 7.13 mmol/L; LDL 3.48 mmol/L	BMI 28.09 Kg/㎡; waistline 101 cm	91/147 mmHg	HbA1c 7.6%; FBG 8.85 mmol/L	NO
S15	male	47	TC 5.80 mmol/L; TG 4.80 mmol/L; LDL 3.45 mmol/L	BMI 29.39 Kg/㎡; waistline 92 cm	102/162 mmHg	FBG 7.38 mmol/L	NO
S16	male	50	TG 2.21 mmol/L	BMI 31.60 Kg/㎡; waistline 91 cm	109/167 mmHg	NO	NO
S17	female	61	TC 6.51 mmol/L; TG 3.58 mmol/L; LDL 4.09 mmol/L	BMI 33.33 Kg/㎡; waistline 100 cm	87/163 mmHg	NO	366.3 μmol/L
S18	female	57	TG 1.58 mmol/L	BMI 31.64 Kg/㎡; waistline 95 cm	90/175 mmHg	NO	NO
S19	female	55	TC 8.08 mmol/L; TG 2.15 mmol/L; LDL 5.16 mmol/L	BMI 26.75 Kg/㎡	104/190 mmHg	NO	NO
S20	male	53	TC 6.35 mmol/L; LDL 4.22 mmol/L	NO	98/167 mmHg	HbA1c 8.0%; FBG 7.87 mmol/L	NO
C1	female	60	NO	NO	NO	NO	NO
C2	male	58	NO	NO	NO	NO	NO
C3	female	59	NO	NO	NO	NO	NO
C4	male	58	NO	NO	NO	NO	NO
C5	female	55	NO	NO	NO	NO	NO
C6	female	59	NO	NO	NO	NO	NO
C7	female	40	NO	NO	NO	NO	NO
C8	female	61	NO	NO	NO	NO	NO
C9	male	56	NO	NO	NO	NO	NO
C10	male	61	NO	NO	NO	NO	NO
C11	female	63	NO	NO	84/145 mmHg	NO	NO
C12	male	63	NO	NO	NO	NO	NO
C13	male	65	NO	NO	NO	NO	NO
C14	male	65	NO	NO	77/150 mmHg	NO	NO
C15	male	57	NO	NO	NO	NO	NO
C16	female	57	NO	NO	96/149 mmHg	NO	NO
C17	male	64	NO	NO	NO	NO	NO
C18	male	49	NO	NO	NO	NO	NO
C19	female	62	NO	NO	NO	NO	NO
C20	female	30	NO	BMI 24.7 Kg/㎡	NO	NO	NO

**Table 2.** Non-alcoholic fatty liver disease in participants.

Personnel number	Liver function outliers	History of smoke (average)	History of alcohol use	Fatty Liver diagnose by abdominal ultrasound	Viral hepatitis or other liver disease
S1	TBil 46.0 μmol/L; ALT 41 U/L	Never	<1/month	Severe fatty liver	NO
S2	NO	Never	<1/month	Severe fatty liver	NO
S3	NO	40/day	5 g/day	Severe fatty liver	NO
S4	NO	Never	<1/month	Severe fatty liver	NO
S5	TBil 22.1 μmol/L	20/day	2 g/day	Severe fatty liver	NO
S6	TBil 21.1 μmol/L; ALT 46 U/L	50/day	<1/month	Severe fatty liver	NO
S7	TBil 21.8 μmol/L	Never	<1/month	Severe fatty liver	NO
S8	NO	Never	<1/month	Severe fatty liver	NO
S9	ALT 49 U/L	40/day	2 g/day	Severe fatty liver	NO
S10	ALT 46 U/L	Never	<1/month	Severe fatty liver	NO
S11		20/day	<1/month	Severe fatty liver	NO
S12	ALT 43 U/L	40/day	4 g/day	Severe fatty liver	NO
S13	TBil 31.0 μmol/L; AST 46 U/L	Never	<1/month	Severe fatty liver	NO
S14	ALT 45 U/L	20/day	2 g/day	Severe fatty liver	NO
S15	NO	30/day	5 g/day	Severe fatty liver	NO
S16	NO	Never	<1/month	Severe fatty liver	NO
S17	NO	Never	<1/month	Severe fatty liver	NO
S18	TBil 20.3 μmol/L	Never	<1/month	Severe fatty liver	NO
S19	NO	Never	<1/month	Severe fatty liver	NO
S20	TBil 21.2 μmol/L; AST 51 U/L	20/day	4 g/day	Severe fatty liver	NO
C1	NO	Never	<1/month	NO	NO
C2	NO	10/day	2 g/day	NO	NO
C3	NO	Never	<1/month	NO	NO
C4	NO	20/day	2 g/day	NO	NO
C5	NO	Never	<1/month	NO	NO
C6	NO	Never	<1/month	NO	NO
C7	NO	Never	Less than once a month	NO	NO
C8	NO	Never	<1/month	NO	NO
C9	NO	5/day	2 g/day	NO	NO
C10	NO	20/day	2 g/day	NO	NO
C11	NO	Never	<1/month	NO	NO
C12	NO	40/day	2 g/day	NO	NO
C13	NO	20/day	2 g/day	NO	NO
C14	NO	male	2 g/day	NO	NO
C15	NO	20/day	5 g/day	NO	NO
C16	NO	Never	<1/month	NO	NO
C17	NO	10/day	2 g/day	NO	NO
C18	NO	Never	<1/month	NO	NO
C19	NO	Never	<1/month	NO	NO
C20	NO	Never	<1/month	NO	NO

Considering the crucial impact of the gut microbiota on the hepatic immune system, there is a strong possibility that the gut microbiota plays an indispensable role in the immunological tolerance of NASH. Hence, 16S rRNA sequencing was conducted to identify the features of the gut microbiome of both groups and screen the contributing species. The Shannon index showed no statistical difference between the healthy and NASH groups (Figure 1e). Likewise, microbial coenosis showed only a slight difference between the two groups and was almost indistinguishable (Figure 1f), which could be explained by the similar dietary habits brought up from the same geographical location.

We then compared the differences in both phylum and species levels between the two groups (Figure 1g,h). At the phylum level, the abundance of *Firmicutes* and *Proteobacteria* was substantially higher in the NASH group. In contrast, the abundance of *Bacteroides* markedly decreased in the NASH group. At the species level, the abundance of *B. uniformis*, *Bifidobacterium longum*, *Parabacteroides distasonis*, *Bilophila*, *B. bifidum* was significantly higher in the healthy group than in the NASH group. The species that best interpreted the healthy and NASH groups are shown in Figure 1i. *B. uniformis* and *B. bifidum* were enriched in the healthy group.

To further identify the contributing gut microbiota, we chose two species with maximum differences between NASH and healthy groups, *B. uniformis* and *B. bifidum*, for further experiments. Purchased strains were cultured and verified by 16S rRNA sequencing (Supplementary Figure S1a,b). The predicted COG functions of *B. uniformis* and *B. bifidum* are shown in Supplementary Figure S1c. Function prediction of KEGG associated with metabolic disorders and immunity of *B. uniformis* and *B. bifidum* are shown in Supplementary Table S2.

### *B. uniformis* and *B. bifidum* averted liver dysfunction and metabolic disorders in HFD induced NASH mice

**Figure 2.**
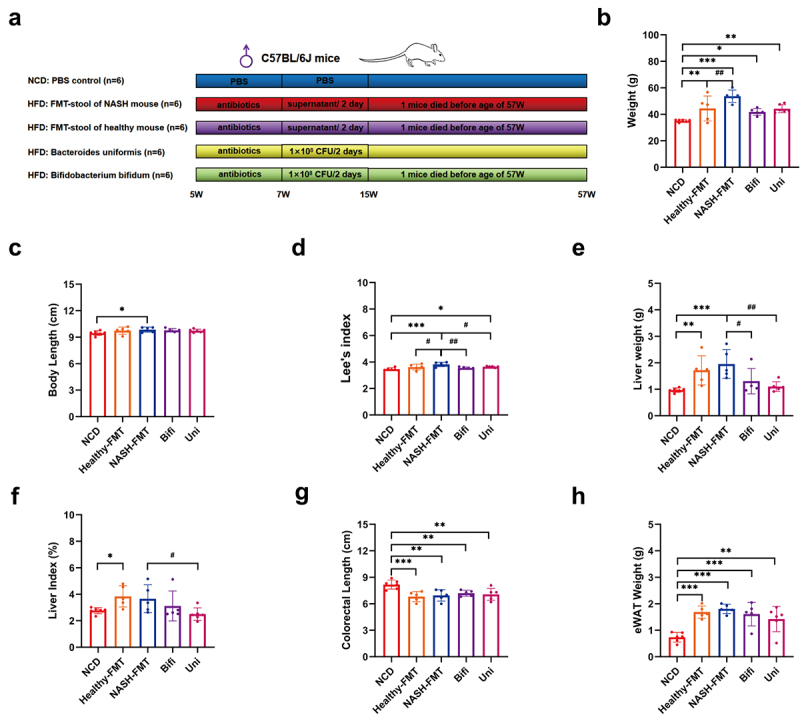
*B. uniformis* and *B. bifidum* showed anti-obesity effects on HFD-induced NASH mice. (a) Schematic diagram showing the experimental design, and timeline of the animal model. (b) Weight of mice in different groups. (c) Body length of mice in different groups. (d) Lee’s’s index of mice in different groups. (e) Liver weight of mice in different groups. (f) Liver index of mice in different groups. (g) Length of the colon and rectum of mice in different groups. (h) Weight of eWAT of mice in different groups. Compared with the NCD group, *p < .05, **p < .01, ***p < .001; compared with the NASH-FMT group, ^#^*p* < .05, ^##^*p* < 0.01, ^###^*p* < 0.001.

After establishing a pseudo-sterile mouse model using a combination of antibiotics, fecal bacteria derived from HFD-induced NASH mice and NCD-fed mice of the same coevality, *B. uniformis* and *B. bifidum*, were transplanted (Figure 2a). Compared to the NCD group, mice exhibited obvious obesity after 52 weeks of HFD, as reflected by substantially elevated body weight (Figure 2b), and the NASH-FMT group showed an increase in body length compared to the NCD group (Figure 2c). In addition, compared to the NCD group, Lee’s index and liver weight in the NASH-FMT group mice increased, and the administration of *B. uniformis* and *B. bifidum* reversed these changes (Figure 2d–f). Compared to the NCD group, the colorectal length and eWAT weight of each group fed a high-fat diet showed a decreasing and increasing trend, respectively (Figure 2g,h).

**Figure 3.**
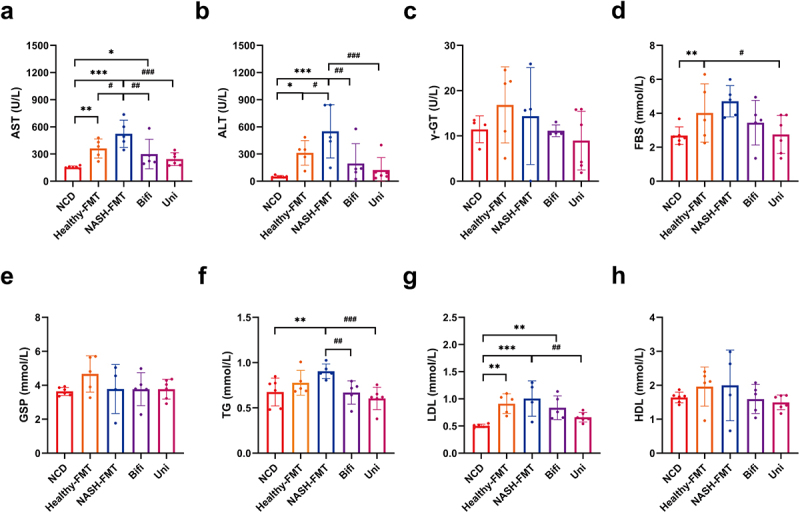
*B. uniformis* and *B. bifidum* improved liver damage and dyslipidemia in HFD-induced NASH mice. (a) AST of mice in different groups. (b) ALT of mice in different groups. (c) γ-GT of mice in different groups. (d) Fasting blood glucose of mice in different groups. (e) Glycated serum protein of mice in different groups. (f) Triglycerides of mice in different groups. (g) LDL of mice in different groups. (h) HDL of mice in different groups. Compared with the NCD group, *p < .05, **p < 0.01, ***p < .001; compared with the NASH-FMT group, ^#^*p* < .05, ^# #^*p* < .01, ^# # #^*p* < .001.

Subsequently, we examined the biochemical profiles of the blood in each group (Figure 3). A long-term HFD caused liver dysfunction and disturbance of glucose and lipid metabolism, as reflected by elevated serum AST, ALT, FBS, TG and LDL, and was more pronounced in the Healthy-FMT and NASH-FMT groups. Both *B. uniformis* and *B. bifidum* transplantation significantly reduced the elevated serum AST, ALT, and TG levels in the NASH-FMT group, and *B. uniformis* transplantation additionally lowered serum LDL.

### *B. uniformis* and *B. bifidum* mitigated liver lesions while maintaining integrity of intestinal mucosal barrier in HFD induced NASH mice

**Figure 4.**
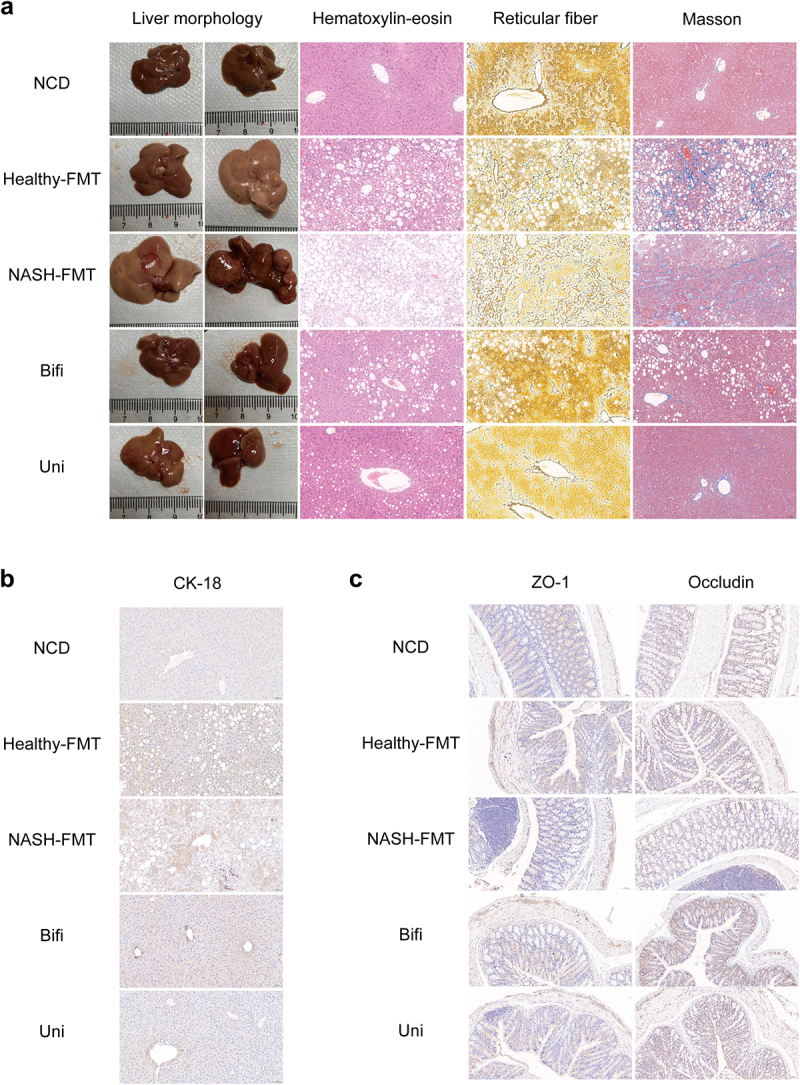
*B. uniformis* and *B. bifidum* improved liver histopathology and the intestinal mucosal barrier in HFD-induced NASH mice. (a) Representative images of liver morphology, H&E, reticular fiber and Masson staining of mice in different groups. Bar: 50 μm. (b) Representative images of immunohistochemistry of hepatic CK-18. Bar: 50 μm. (c) Representative images of immunohistochemistry of colorectal occludin and ZO-1. Bar: 50 μm.

After long-term HFD, mice in the NASH-FMT group developed NASH and even liver cancer (Figure 4a,b). H&E staining revealed noticeable changes in hepatocytes, including ballooning, steatosis, inflammation, and fibrosis, in the NASH-FMT and Healthy-FMT groups (Figure 4b). As expected, *B. uniformis* and *B. bifidum* significantly attenuated all these pathological changes, particularly *B. uniformis* (Figure 4a,b). To elucidate the impact of long-term HFD and probiotic treatment on the intestinal mucosal barrier, we detected ZO-1 and occludin, two typical intestinal tight junction proteins, in each group using immunohistochemistry. The results showed that compared to the NASH-FMT group, *B. uniformis* and *B. bifidum* reversed colorectal inflammation and the declined occludin levels (Figure 4c).

### Immune dysfunction exists in the NASH model induced by a high-fat diet, with *B. uniformis* and *B. bifidum* reversing liver NK cell-mediated immune tolerance

**Figure 5.**
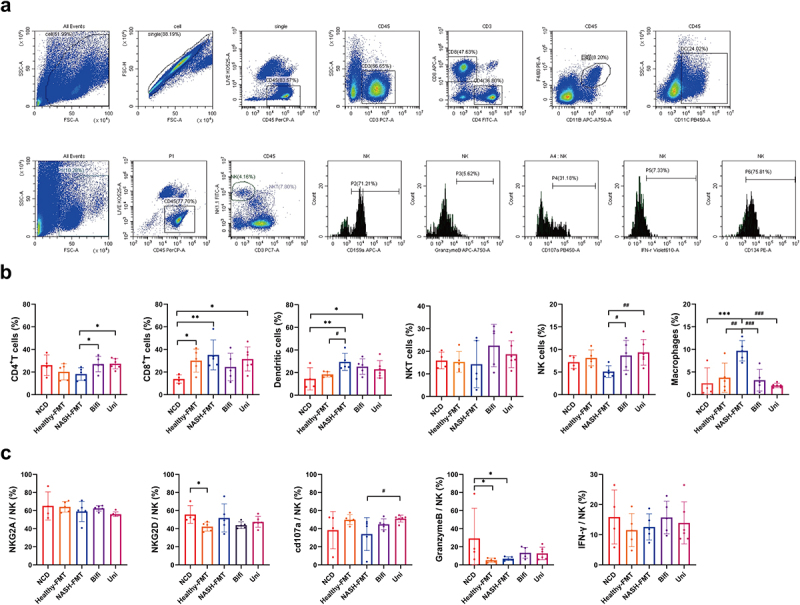
Hepatic NK cell immune intolerance was improved by *B. uniformis* and *B. bifidum* in HFD-induced NASH mice. (a) Gating strategy for analysis of hepatic immune cell infiltration. (b) Hepatic immune microenvironment by flow cytometry. (c) Functional molecular markers on hepatic NK cells by flow cytometry. Compared with the NCD group, *p < .05, **p < .01, ***p < .001; Compared with the NASH-FMT group, ^#^*p*<0.05, ^# #^*p* < .01,^# # #^*p* < .001.

To further explore the hepatic immune status in NASH mice and clarify the functional significance of *B. uniformis and B. bifidum* in maintaining hepatic immune homeostasis, we examined the proportion of immune cells and hepatic NK cell function using flow cytometry (Figure 5a). Although the decreases in CD4^+^T cells, NKT cells, and NK cells showed no statistical difference between the NCD and NASH-FMT groups, the numbers of CD8^+^T cells and dendritic cells (DCs) were significantly increased in the NASH-FMT group (Figure 5b). Macrophage infiltration was also increased in the NASH-FMT group, indicating aggravated liver inflammation.^18^ Compared with the NASH-FMT group, *B. uniformis and B. bifidum* transplantation resulted in an increased number of hepatic CD4^+^T cells and NK cells, and a decreased number of macrophages (Figure 5b).

Considering the critical role of NK cells in the occurrence and progression of NASH, we examined surface markers of hepatic NK cells. The expression of the inhibitory receptor NKG2A was not statistically different among the five groups. However, compared to the NCD group, the activating receptors NKG2D and Granzyme B were statistically decreased in the Healthy-FMT group, and granzyme B was statistically decreased in the NASH-FMT group (Figure 5c). For the other activating receptor, IFN-γ, although there was a downward trend in the Healthy-FMT and NASH-FMT groups, there was no statistical difference (Figure 5c). Both *B. uniformis and B. bifidum* transplantation showed an upward trend in the number of CD107a, Granzyme B and IFN-γ. Disappointingly, only *B. uniformis* transplantation statistically upregulated CD107a, as a result of the insufficient sample size and excessive individual differences in each group.

To better clarify whether hepatic immune tolerance of NK cells was established in NASH mice, we re-detected the proportion of immune cells and the effector factors of hepatic NK cells in NCD fed mice, HFD induced NASH mice, *B. uniformis* treated and *B. bifidum* treated NASH mice (Supplementary Figure S2). Notably, this result was further confirmed by the statistically decreased number of NK cells, NKG2D and CD107a in the NASH group. As expected, increased colonization of *B. uniformis* and *B. bifidum* by 8 weeks of exogenous supplementation (Supplementary Figure S3), enhanced the cytotoxicity of NK cells by statistically increasing NKG2D and the number of NK cells, respectively.

### *B. uniformis* and *B. bifidum* regulated activating and inhibitory signals of NK cell to resist hepatic immune tolerance

**Figure 6.**
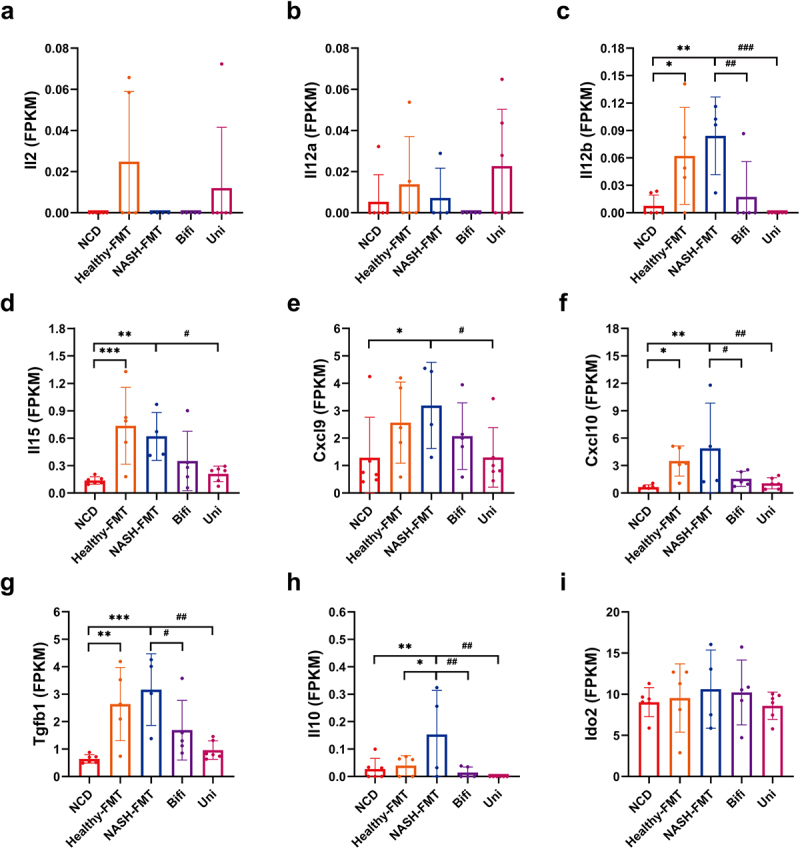
Hepatic enhancement and inhibitory signals of NK cells in HFD-induced NASH mice. (a) Hepatic *Il2* of mice in different groups. (b) Hepatic *Il12a* of mice in different groups. (c) Hepatic *Il12b* of mice in different groups. (d) Hepatic *Il15* of mice in different groups. (e) Hepatic *Cxcl9* of mice in different groups. (f) Hepatic *Cxcl10* of mice in different groups. (g) Hepatic *Tgfb1* of mice in different groups. (h) Hepatic *Il10* of mice in different groups. (H) Hepatic *Il10* of mice in different groups. (i) Hepatic *Ido2* of mice in different groups. Compared with the NCD group, *p < .05, **p < .01, ***p < .001; compared with the NASH-FMT group, ^#^*p* < .05, ^# #^*p* < .01, ^# # #^*p* < .001.

Recently, the interaction between the dynamic microenvironment and NK cells has received increasing attention,^19^ and the specialized hepatic microenvironment influenced by HFD has resulted in NK cell dysfunction and insufficient infiltration. To explore the mechanism by which NK cells crosstalk with the hepatic microenvironment, hepatic transcriptome analysis was conducted. We produced KEGG scanners and volcano plots containing the top 20 genes (Supplementary Figure S4). Compared to the NCD group, 75 genes in the focal adhesion pathway (KO 04510), including *Spp1*, *Col6a2*, *Rac2*, *Vav1*, *Pak1* and Thbs4, were increased in the NASH-FMT group, whereas *Mylk3* was decreased in the NASH-FMT group. Notably, the above genes were reversed by *B. uniformis* and *B. bifidum* transplantation compared to those in the NASH-FMT group. Despite undifferentiated statistics, *Ptk2* (*Fak*), one of the core genes in the focal adhesion pathway, exhibited a downward trend in the Uni and Bifi groups compared to the NASH-FMT group. We then quantified the biologically activating and inhibitory signaling factors that regulate NK cells (Figure 6). Although the activating factors *Il2*, *Il12b*, *Il15*, *Cxcl9*, and *Cxcl10* of NK cells showed upregulated trend in the NASH-FMT group (Figure 6a–f), the inhibitory factors *Tgfb1*, *Il10*, and *Ido2* also showed upregulated trend (Figure 6g–i), which may be one of the reasons why it leads to NK cell immune tolerance. *B. uniformis* and *B. bifidum* reversed the activation of NK cell inhibitory receptors by NASH, which was consistent with the restoration of NK cell function.

To further understand the mechanism of hepatic NK cell immune tolerance in HFD-induced NASH mice, hepatic proteome analysis was conducted, and the top 20 upregulated and downregulated KEGG pathway enrichments are shown in Supplementary Figure S5. Compared with that in the NCD group, the focal adhesion pathway and TNF signaling pathway were significantly altered, and the NAFLD pathway was significantly upregulated in the NASH-FMT group and was statistically reversed by *B. uniformis* and *B. bifidum* (not in the top 20 pathways) transplantation (Supplementary Figure S5). Moreover, the protein expression profiles in the natural killer cell-mediated cytotoxicity pathway also hinted at the reduced activity of hepatic NK cells in the NASH-FMT group, including downregulated NKG2AB, NKGP46, NKG2DL, IFN-γ, granzyme, TNF-α, and perforin (Supplementary Figure S6).

**Figure 7.**
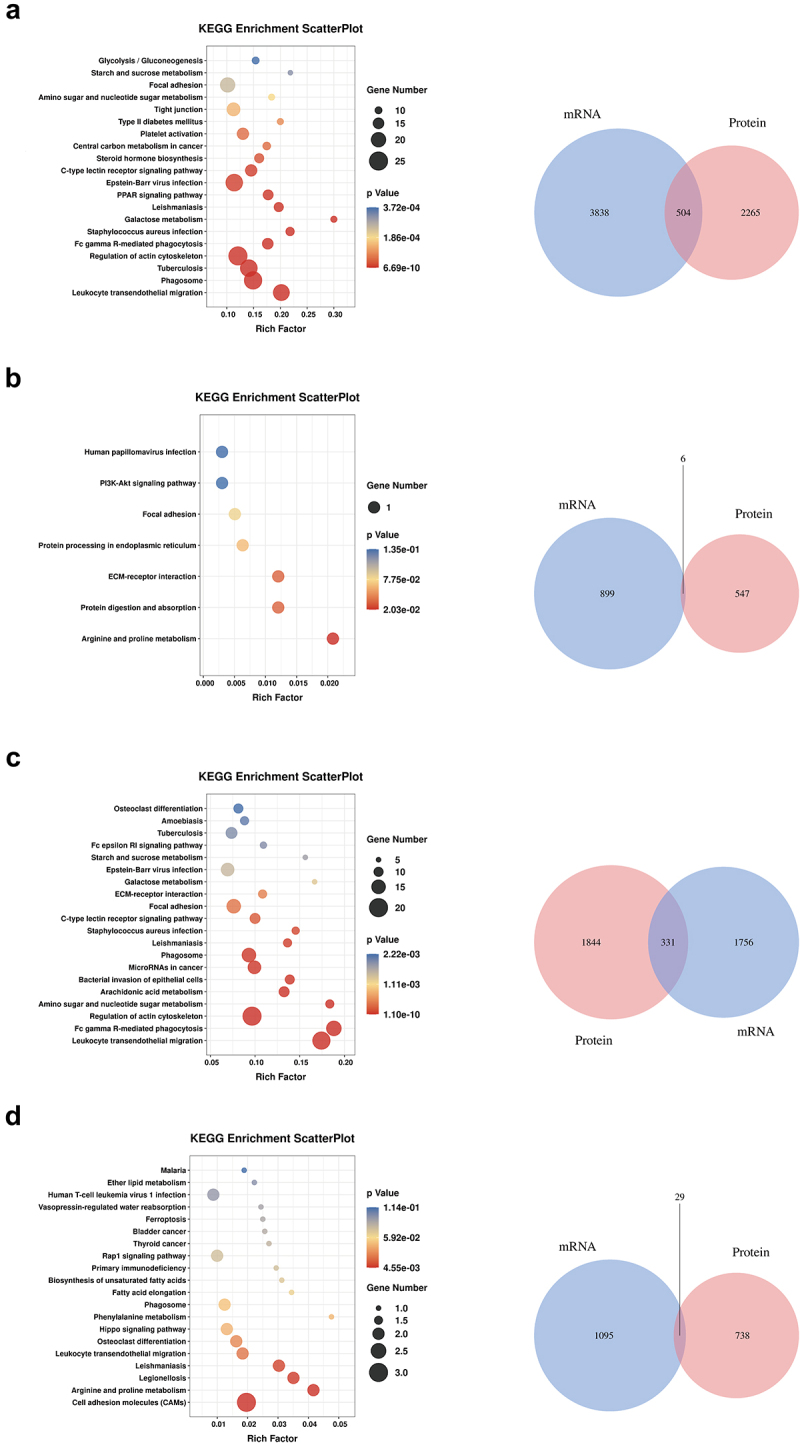
KEGG pathway enrichments and venn diagrams by liver proteomics and RNA sequencing co-analysis. (a) KEGG enrichment scatter plot and venn diagram between the NASH-FMT group and the NCD group. (b) KEGG enrichment scatter plot and venn diagram between the NASH-FMT group and the Healthy-FMT group. (c) KEGG enrichment scatter plot and venn diagram between the NASH-FMT group and the uni group. (d) KEGG enrichment scatter plot and venn diagram between the NASH-FMT group and the Bifi group. KEGG enrichment details were shown in supplementary materials.

Next, a comprehensive analysis of the transcriptome and proteomics was conducted, and the changes in both proteins and genes and KEGG pathway enrichments are displayed in Figure 7. Compared with the NCD group, the focal adhesion pathway was significantly activated in the NASH-FMT group (Figure 7a). Overactive activity of the focal adhesion pathway was blunted in both the Healthy-FMT and Uni groups (Figure 7b,c). In addition, the ECM-receptor interaction pathway was activated in the NASH-FMT group and restored by *B. uniformis* transplantation (Figure 7b,c). Notably, compared to the NCD group, both the gene and protein levels of *Cd44* and *Cd93* were significantly enhanced in the NASH-FMT group and restored in the Uni group. These results collectively indicated that there were multiple outlooks of which *B. uniformis* attenuated hepatic immune tolerance.

### Fecal metagenome analysis in different groups

To further clarify the changes in gut microbiota among the different groups and confirm the colonization of *B. uniformis* and *B. bifidum* (Supplementary Figure S7), we conducted metagenomic analysis of the gut microbiota in each group of mice. The results showed no difference in the Shannon index at the species level between the groups (Supplementary Figure S8a). The Venn diagram further illustrates the number of common and differential bacteria in each group (Supplementary Figure S8b). In addition, in the principal coordinates analysis, compared to the NASH-FMT and Healthy-FMT groups, the species distribution similarity between the Uni and NCD groups was higher, and that between the Bifi and NCD groups was the highest (Supplementary Figure S8c). We also compared the six differential phyla with the highest abundance and 10 bacterial species with the highest abundance in the feces of the mice in each group (Supplementary Figure S8d,e). The results showed that compared to the healthy control group, the proportion of Firmicutes to Bacteroidetes in the NASH-FMT group decreased significantly. In contrast, the ratio of Firmicutes to Bacteroidetes in the gut microbiota of the *B. uniformis* and *B. bifidum* groups was higher than that in the NASH-FMT group. Subsequently, we analyzed changes in the KEGG pathway (LEVEL 3) (Supplementary Figure S8f). The results showed that carbon metabolism, glycosaminoglycan degradation, and oxidative phosphorylation were enriched in the NASH-FMT group. Flagellar assembly, Bacterial chemotaxis, Legionetaxis, and NOD-like receptor signaling pathway were abundant in the Bifi group, whereas counterfeit metabolism, inositol phosphate metabolism, and sulfur relay system were enriched in the Uni group. In addition, we constructed LDA histograms at the species level and a correlation network analysis of specifications and KEGG functions (Supplementary Figure S8g,h), showing the types of microorganisms （such as Mucispirillum schaedleri and Siphoviridae sp.）with significant impact in each group and the correlation between gut microbiota and metabolic pathways. Intriguingly, we found that Lichtheimia ramosa, an opportunistic fungal pathogen mostly occurring in immunocompromised organisms,^20^ was significantly increased in the NASH-FMT group compared with the NCD group and the application of *B. uniformis and B. bifidum* significantly reduced its level compared to the NASH-FMT group (Supplementary Figure S9).

## Discussion

NASH is a precursor of liver fibrosis and cirrhosis, posing a significant health burden.^6^ NK cells play a pivotal role in the ongoing progression of NASH. However, the role of NK cells in NASH remains controversial.^21^ In our study, patients with more than 10 years of severe NAFLD as well as those with at least two types of metabolic disorders were enrolled in the NASH group. Previous studies have shown that CK18 is a potent tool for the noninvasive diagnosis of NASH,^22,23^ and the specificity of cCK18 for NASH prediction is higher than that of M2BPGi, FIB4 index, APRI, and other diagnostic indicators.^24^ Thus, we examined the serum CK-18 levels in both groups, and our results were consistent with those of published studies, manifesting as a significantly increased level of serum CK18 in the NASH group.

Although a wealth of studies have accumulated on the immunopathogenesis of NASH, there has been an increase in IL-6 and IFN-γ^8^ due to the over-activation of hepatic immune cells, accompanied by the downregulation of IL-10.^25^ Interestingly, in our study, we observed a statistically significant increase in serum IL-10 levels and non-statistical decreases in serum IFN-γ and IL-6 levels in the NASH group. Although there was a lack of flow cytometry analysis, these atypical changes potentially supported the concept and possibility of peripheral immune tolerance, and even hinted at immune tolerance in the liver. Our follow-up study showed that mice fed a HFD to induce NASH accumulated a statistically higher number of DCs, macrophages, and CD8^+^T cells, and a non-statistically lower number of NK cells (particularly activated NK cells), CD4^+^T cells and NKT cells in the liver. Although there was no statistical reduction in cd107a, and IFN-γ in the NASH-FMT group, presumably owing to excessive discrepancy within the group and insufficient sample size, the reduction in Granzyme B showed a statistical difference. Remedially, the result was thereafter confirmed by the statistically decreased number of NK cells, NKG2D and CD107a in the NASH group. Our results are inconsistent with those of NASH mice induced by MCD, CDHFD, and those injected with streptozotocin.^8^ Thus, we speculate that the immunopathogenesis of NASH induced by various diets may be distinct.

The gut microbiota plays a key role in the infiltration and activation of NK cells.^19^ Some of them enhance NK cell infiltration and activity by promoting the secretion of inflammatory factors,^26,27^ such as *Lactobacillus plantarum*,^27^ and others dampen NK cell infiltration and activity.^28^ To further clarify whether gut microbiota could intervene in the progression of NASH and regulate NK cells, we screened two potential probiotics that were depleted in NASH patients and transplanted them into a HFD-induced NASH model. The results showed that *B. uniformis* and *B. bifidum* not only improved the pathological and biochemical changes, but also enhanced the infiltration and activation of hepatic NK cells, further suggesting that *B. uniformis* and *B. bifidum* may improve NK cell immune tolerance in the liver, thereby improving the progression of NASH. Thus, clarifying the underlying mechanisms may provide new directions for immune cell-based therapies for NASH.

Studies have shown that the function of NK cells is affected by various microenvironments, including the immune microenvironment, microbial microenvironment, and metabolic microenvironment,^19^ and the functional transformation of NK cells depends on the balance between enhanced and inhibitory signals.^29^ We analyzed the enhanced molecules, including *IL-15*, *CXCL-9*, *CXCL-10*, *IL-2* and *IL-12*, and inhibitory molecules, including *IL-10*, *TGF-β* and *IDO*,^19^ and found that both activating and inhibitory molecules of NK cells showed upregulated trends in HFD-induced NASH mice, and both *B. uniformis* and *B. bifidum* reduced hepatic *IL-10* and *TGF-β1*. Notably, *B. uniformis* and *B. bifidum* appeared to be surprising activating agents of hepatic NK cells, manifesting as increased infiltration of NK cells, and statistical upregulation of Granzyme B by prophylactic use of *B. uniformis*, as well as statistically increasing NKG2D and infiltration of NK cells by afterward supplementation of *B. uniformis* and *B. bifidum*, respectively. These results collectively indicate that *B. uniformis* and *B. bifidum* could resist hepatic NK cell dysfunction in NASH, at least by reducing the inhibitory molecules of NK cells.

In addition to the functional signals of NK cells, prophylactic treatment with *B. uniformis* and *B. bifidum* also restored transcriptional changes in the focal adhesion pathway. Compared to the NCD group, multiple genes in the focal adhesion pathway were statistically upregulated in the NASH-FMT group, including immune-mediated genes such as *Spp1*, *Col6a2* and *Rac2*.^30–32^ *Col6a2* is related to the tumor immune microenvironment and is negatively correlated with activated NK cells.^31^ *Rac2*, a functional molecule that participates in the maturation and migration of NK cells, is also a negative regulator of NK cells, manifesting as reducing natural cytotoxic receptor (NCR) NKp 46, NKp 30, and activated receptor CD16, as well as enhancing the inhibitory receptor NKG 2A and its corresponding activated receptor NKG 2C.^32^ All the aforementioned genes were statistically reduced by *B. uniformis* and *B. bifidum*, which may partially explain how *B. uniformis* and *B. bifidum* attenuate the reduction in hepatic NK cell infiltration and activation. *Fak*, one of the core genes in the focal adhesion pathway, was found to downregulate the primary ligand MICA of NKG2D immune receptors through the *Fak/Src* pathway, thereby weakening susceptibility to NK cell-mediated killing.^33^ In our study, it showed non-statistical increment in the NASH-FMT group and non-statistical reduction in the Uni-FMT and Bifi-FMT groups.

Additionally, hepatic *CD44* (gene in ECM-receptor interaction pathway) and *CD93* were higher in the NASH-FMT group than in the NCD group, suggesting another possible mechanism for NK cell immune tolerance in NASH. *CD44* can antagonize NK cell internalization adhesion by reducing N-cadherin-mediated intercellular adhesion, inhibiting cell formation, and weakening NK cell cytotoxicity against tumors.^34^ Both anti-*CD44* antibody and *CD44* deficiency in mice mitigated liver steatosis, attenuated adipose inflammation, and improved glucose tolerance in diet-induced obese mice.^35,36^ *CD93* is also involved in NK cell immune tolerance and is negatively correlated with NK cells,^37^ and anti-*CD93* antibodies can increase the infiltration of NK cells.^38^ In our study, *B. uniformis* transplantation statistically reduced the expression of hepatic *CD44* and *CD93*, offering another considerable mechanism by which it enhances NK cell infiltration and activation.

Fungi may also play essential biological functions in immune responses. In particular, *Monascus purpureus* supplementation ameliorates NAFLD induced by high-fat, high-fructose, and high-cholesterol diet,^39^ and genistein-stimulated *Monascus purpureus* can enhance beneficial bacteria such as *Lactobacillus*, showing a positive correlation with most immunological indicators.^40^ *Lichtheimia ramosa*, an opportunistic fungal pathogen of the order Mucorales,^41^ was found to be mostly over-proliferated in patients with low immune function.^20^ In our study, fecal *Lichtheimia ramosa* was substantially increased in NASH mice, and was largely eliminated by *B. uniformis* and *B. bifidum*. Since most *Lichtheimia ramosa* infections occur in immunocompromised organisms, our results suggested a possibility of immune tolerance in NASH mice.

Inflammation is an important driver of the immune response.^42^ Studies have reported that progressively accumulating inflammation leads to impaired and exhausted innate and adaptive immunity.^43^ Macrophages, which trigger inflammation, play a prominent role in the initiation and development of NASH^44^ and are also inhibitors of NK cells.^45,46^ Similar phenotypic profiles were observed in our study, manifesting as incremental infiltration of macrophages and attendant NK cell inhibition in the NASH-FMT group (compared to the NCD group). On this basis, it is more persuasive to explain how *B. uniformis and B. bifidum* invigorated NK cells. Prophylactic use of *B. uniformis and B. bifidum* improved all these adverse changes and provided diverse curative according to the foregoing etiology in NASH evolution.

NASH develops primarily due to immune dysfunction and chronic inflammation. This has resulted in awareness that optimal immunotherapy will be a curative treatment for NASH. Regardless of our limitations (absence of histological examination and flow cytometry in participants, insufficient sample size in animal studies, and unverified mechanism and rationale for NK cell immune tolerance in NASH), our data revealed an unreported functional profile of hepatic NK cells in HFD-induced NASH, possibly due to the crosstalk between inflammation, intestinal microecology, and hepatic microenvironment, highlighting that recovery of NK cell function might serve as a candidate target for immunotherapy for NASH. Our findings also imply that targeting intestinal microecology (transplantation of *B. uniformis and B. bifidum*) could bring substantial therapeutic benefits for NASH, especially in invigorating the immune function of hepatic NK cells, and strongly support the preclinical application of *B. uniformis and B. bifidum* to fully harness their potential to target immune function for the treatment of NASH.

## Supplementary Material

Supplemental Material

supplementary material_KEGG enrichment details in fig7.xlsx

## Data Availability

• This study did not report the original code. • The authors confirm that the data supporting the findings of this study are available within the article and its supplementary material. • Any additional information required to reanalyze the data reported in this paper is available from the lead contact upon request.
