# Supplementary material for: Prophylactic treatment with Bacteroides uniformis and Bifidobacterium bifidum counteracts hepatic NK cell immune tolerance in nonalcoholic steatohepatitis induced by high fat diet
Source: Gut Microbes. 2024 Jan 9;16(1):2302065. doi: 10.1080/19490976.2024.2302065 (PMC10793665; doi:10.1080/19490976.2024.2302065)
Supplement: Supplemental Material [file KGMI_A_2302065_SM6317.zip › Table S1.docx]

| **Antibody** | **Cat.no.** | **Company** |
| --- | --- | --- |
| Zombie Aqua™ Fixable Viability Kit | 423101 | Biolegend |
| PerCP/Cyanine5.5 anti-mouse CD45 | 103131 | Biolegend |
| PE/Cyanine7 anti-mouse CD3 | 100219 | Biolegend |
| KIRAVIA Blue 520™ anti-mouse NK-1.1 | 156521 | Biolegend |
| APC anti-mouse CD159a (NKG2AB6) | 142807 | Biolegend |
| PE anti-mouse CD314 (NKG2D) | 130207 | Biolegend |
| Brilliant Violet 421™ anti-mouse CD107a (LAMP-1) | 121618 | Biolegend |
| Brilliant Violet 605™ anti-mouse IFN-γ | 505840 | Biolegend |
| APC/Fire™ 750 anti-human/mouse Granzyme B Recombinant | 372210 | Biolegend |
| Zombie Aqua™ Fixable Viability Kit | 423101 | Biolegend |
| PerCP/Cyanine5.5 anti-mouse CD45 | 103131 | Biolegend |
| PE/Cyanine7 anti-mouse CD3 | 100219 | Biolegend |
| KIRAVIA Blue 520™ anti-mouse CD4 | 100477 | Biolegend |
| APC anti-mouse CD8a | 100712 | Biolegend |
| APC/Cyanine7 anti-mouse/human CD11b | 101225 | Biolegend |
| PE anti-mouse F4/80 | 123109 | Biolegend |
| Brilliant Violet 421™ anti-mouse CD11c | 117329 | Biolegend |

**Supplementary Table 1 Antibodies Used in Multi-Color Flow Cytometry Analysis**
