# Supplementary figures and images for: Prophylactic treatment with Bacteroides uniformis and Bifidobacterium bifidum counteracts hepatic NK cell immune tolerance in nonalcoholic steatohepatitis induced by high fat diet
Source: Gut Microbes. 2024 Jan 9;16(1):2302065. doi: 10.1080/19490976.2024.2302065 (PMC10793665; doi:10.1080/19490976.2024.2302065)

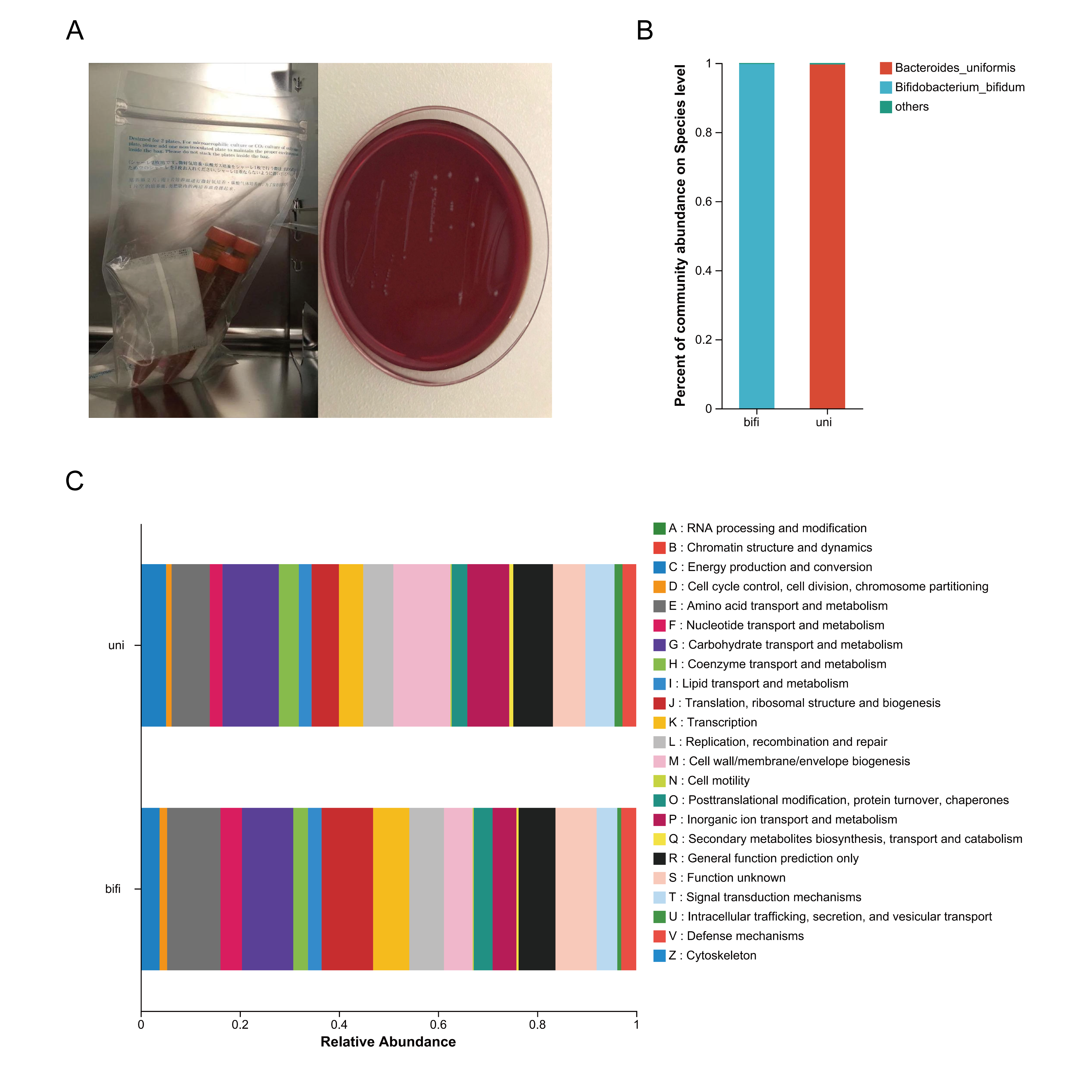

Supplement: Supplemental Material [file KGMI_A_2302065_SM6317.zip › Fig S1.tif]

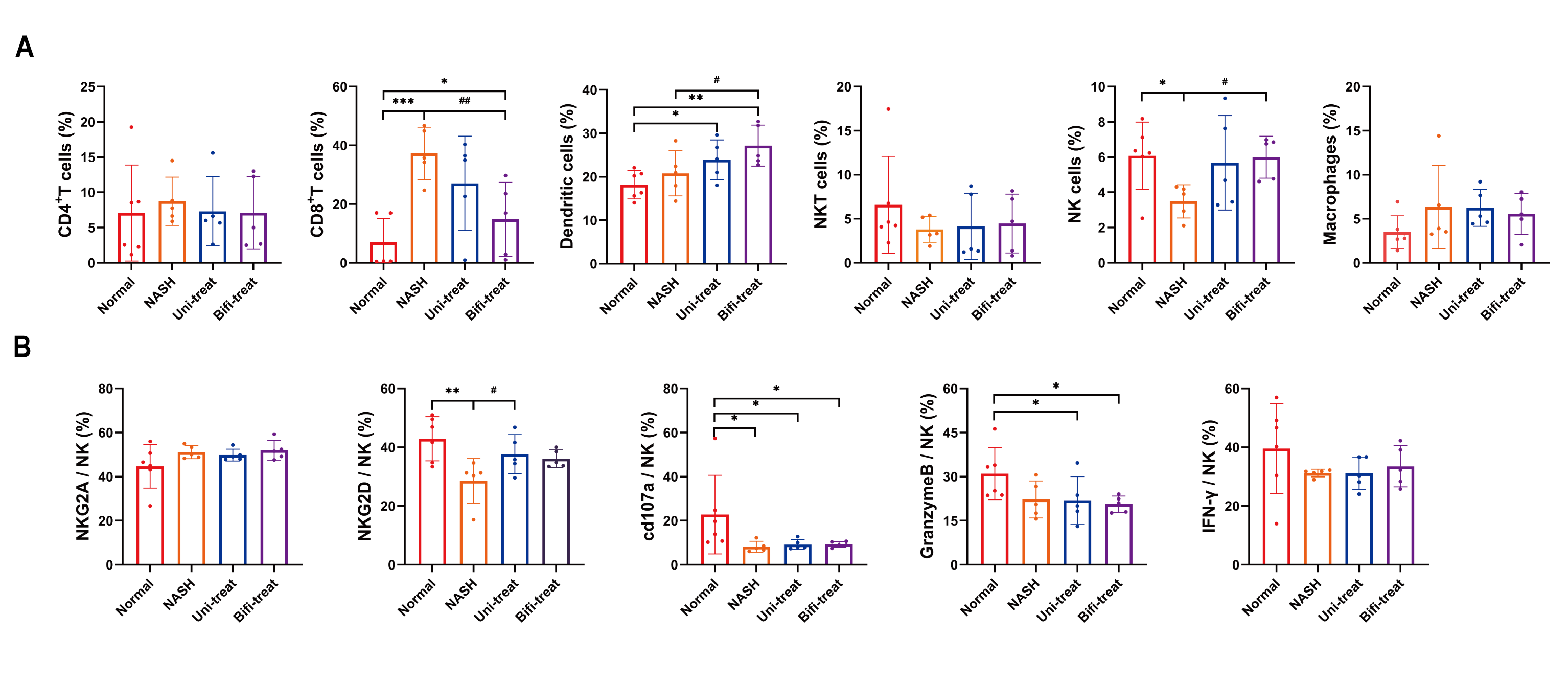

Supplement: Supplemental Material [file KGMI_A_2302065_SM6317.zip › Fig S2.tif]

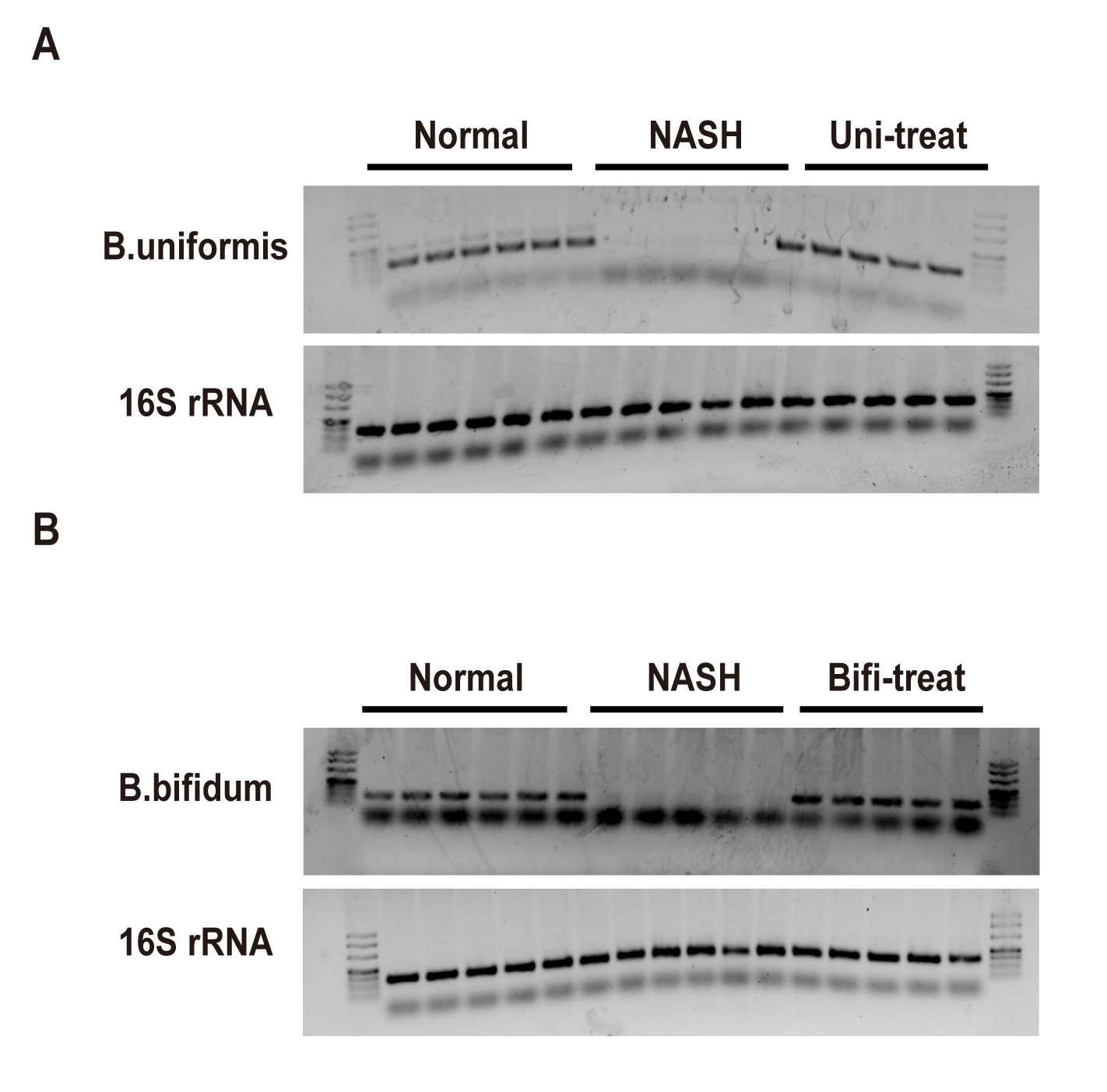

Supplement: Supplemental Material [file KGMI_A_2302065_SM6317.zip › Fig S3.tif]

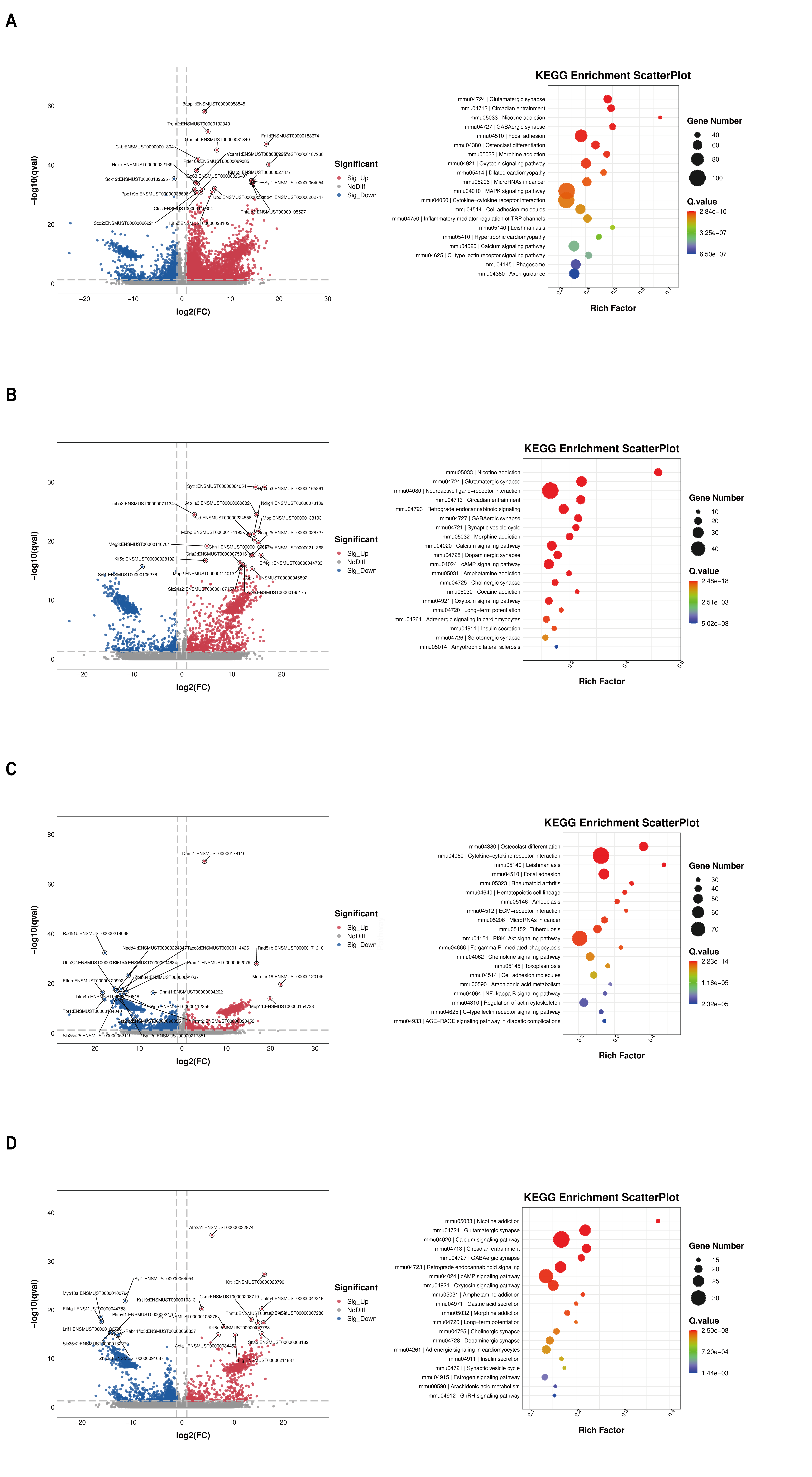

Supplement: Supplemental Material [file KGMI_A_2302065_SM6317.zip › Fig S4.tif]

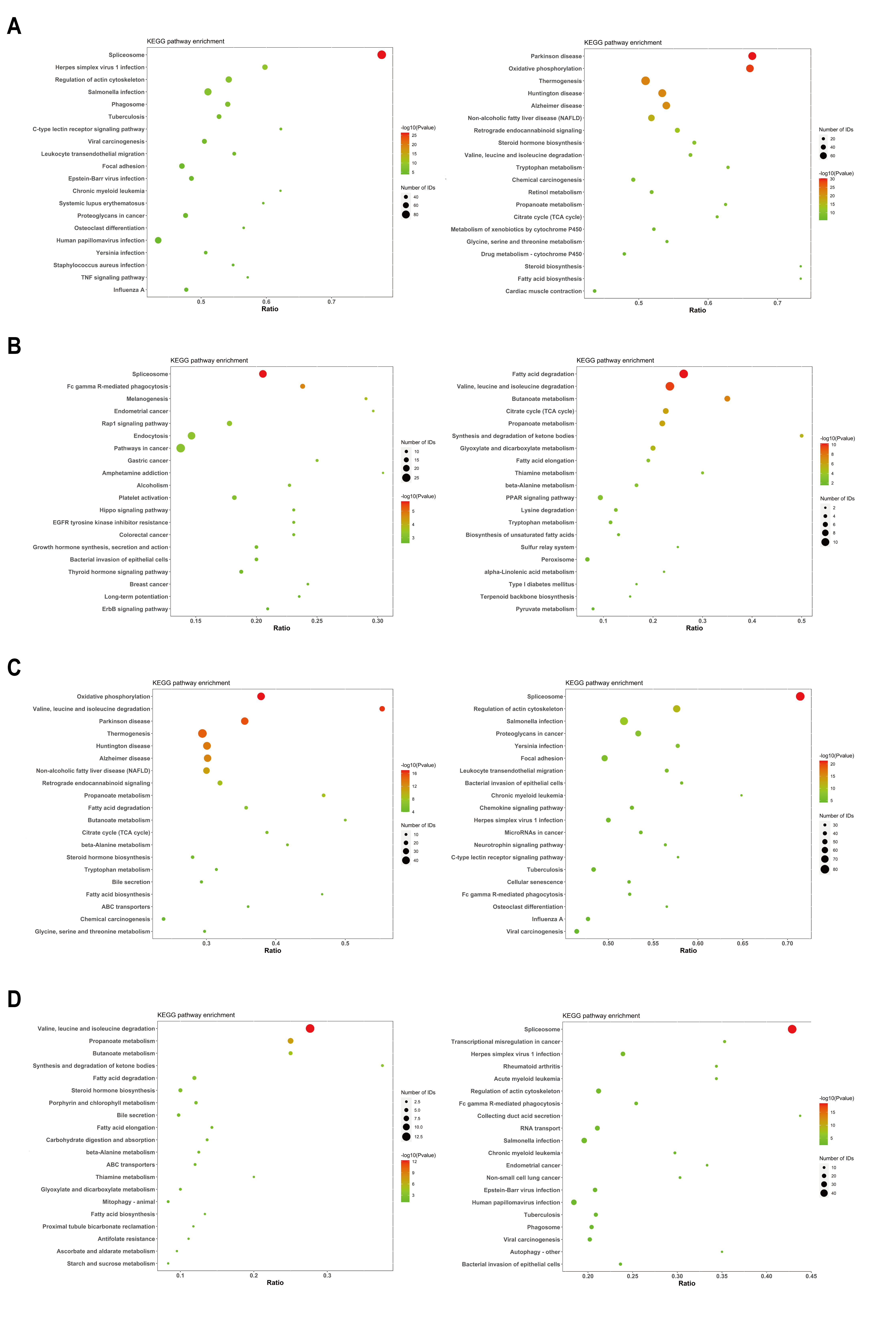

Supplement: Supplemental Material [file KGMI_A_2302065_SM6317.zip › Fig S5.tif]

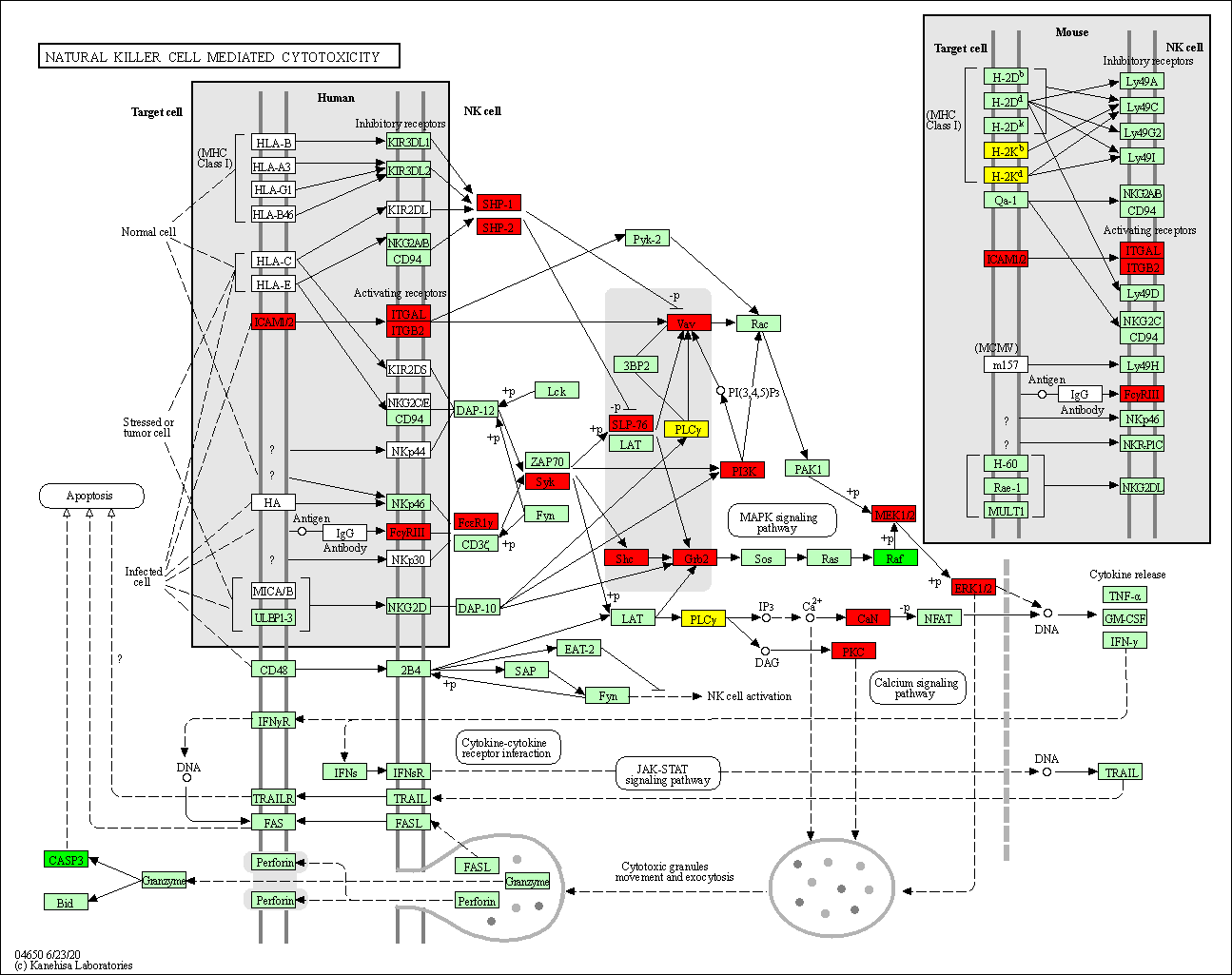

Supplement: Supplemental Material [file KGMI_A_2302065_SM6317.zip › Fig S6.png]

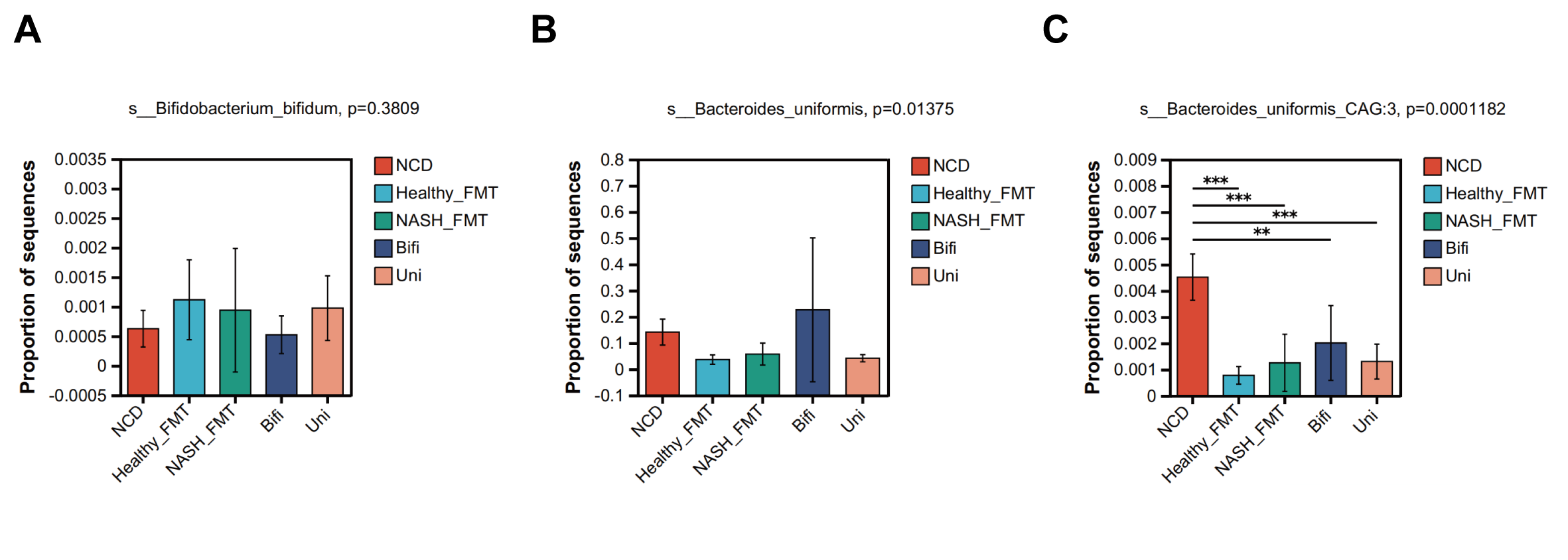

Supplement: Supplemental Material [file KGMI_A_2302065_SM6317.zip › Fig S7.tif]

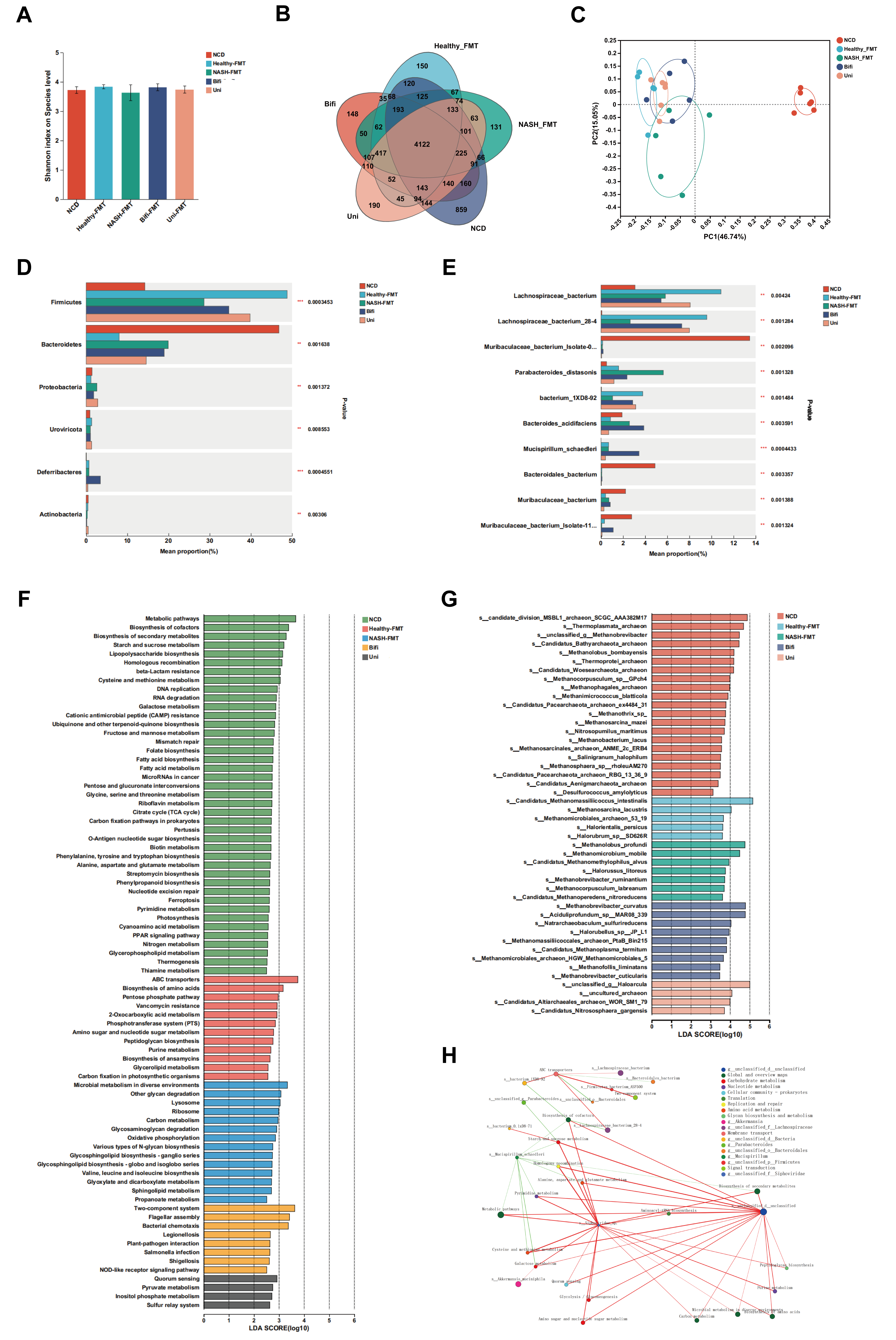

Supplement: Supplemental Material [file KGMI_A_2302065_SM6317.zip › Fig S8.tif]

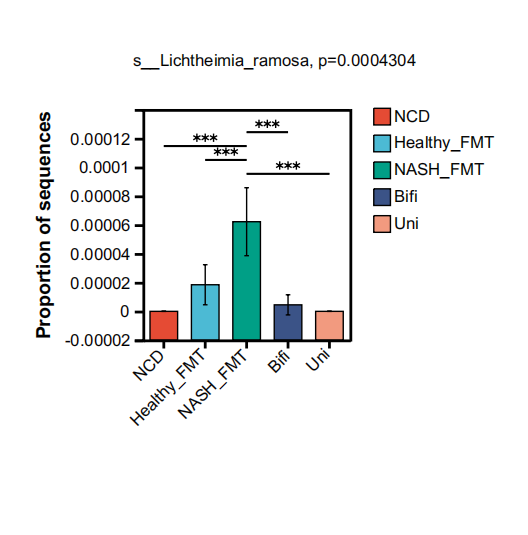

Supplement: Supplemental Material [file KGMI_A_2302065_SM6317.zip › Fig S9.tif]
