# Supplementary material for: Prophylactic treatment with Bacteroides uniformis and Bifidobacterium bifidum counteracts hepatic NK cell immune tolerance in nonalcoholic steatohepatitis induced by high fat diet
Source: Gut Microbes. 2024 Jan 9;16(1):2302065. doi: 10.1080/19490976.2024.2302065 (PMC10793665; doi:10.1080/19490976.2024.2302065)
Supplement: Supplemental Material [file KGMI_A_2302065_SM6317.zip › Table S2.doc]

## Supplementary Table 2 Function prediction of KEGG of *B. uniformis* and *B. bifidum*

| Pathway level 1 | Pathway level 2 | Pathway level 3 | Level 3 Description | Abundance of *Bacteroides uniformis* | Abundance of *Bifidobacterium bifidum* |
| --- | --- | --- | --- | --- | --- |
| Metabolism | Carbohydrate Metabolism | ko00020 | Citrate cycle (TCA cycle) | 232204 | 185049 |
| Metabolism | Carbohydrate Metabolism | ko00010 | Glycolysis / Gluconeogenesis | 366620 | 513690 |
| Human Diseases | Endocrine and Metabolic Disease | ko04940 | Type I diabetes mellitus | 24434 | 20548 |
| Human Diseases | Endocrine and Metabolic Disease | ko04930 | Type II diabetes mellitus | 12219 | 20542 |
| Organismal Systems | Endocrine System | ko04920 | Adipocytokine signaling pathway | 48884 | 102721 |
| Organismal Systems | Endocrine System | ko04910 | Insulin signaling pathway | 24438 | 20551 |
| Organismal Systems | Endocrine System | ko03320 | PPAR signaling pathway | 61171 | 102816 |
| Organismal Systems | Immune System | ko04666 | Fc gamma R-mediated phagocytosis | 1 | 2 |
| Organismal Systems | Immune System | ko04622 | RIG-I-like receptor signaling pathway | 2 | 3 |
| Organismal Systems | Immune System | ko04621 | NOD-like receptor signaling pathway | 12216 | 5 |
| Organismal Systems | Immune System | ko04612 | Antigen processing and presentation | 12216 | 5 |
| Metabolism | Global and Overview Maps | ko01212 | Fatty acid metabolism | 49275 | 185334 |
| Metabolism | Lipid Metabolism | ko01040 | Biosynthesis of unsaturated fatty acids | 12302 | 41177 |
| Metabolism | Lipid Metabolism | ko00600 | Sphingolipid metabolism | 256410 | 143763 |
| Metabolism | Lipid Metabolism | ko00592 | alpha-Linolenic acid metabolism | 23 | 28 |
| Metabolism | Lipid Metabolism | ko00591 | Linoleic acid metabolism | 36668 | 55 |
| Metabolism | Lipid Metabolism | ko00590 | Arachidonic acid metabolism | 18 | 16 |
| Metabolism | Lipid Metabolism | ko00565 | Ether lipid metabolism | 7 | 11 |
| Metabolism | Lipid Metabolism | ko00564 | Glycerophospholipid metabolism | 146636 | 123323 |
| Metabolism | Lipid Metabolism | ko00561 | Glycerolipid metabolism | 61166 | 102793 |
| Metabolism | Lipid Metabolism | ko00140 | Steroid hormone biosynthesis | 48852 | 23 |
| Metabolism | Lipid Metabolism | ko00121 | Secondary bile acid biosynthesis | 24421 | 20537 |
| Metabolism | Lipid Metabolism | ko00120 | Primary bile acid biosynthesis | 24436 | 20557 |
| Metabolism | Lipid Metabolism | ko00100 | Steroid biosynthesis | 12 | 16 |
| Metabolism | Lipid Metabolism | ko00072 | Synthesis and degradation of ketone bodies | 82 | 98 |
| Metabolism | Lipid Metabolism | ko00061 | Fatty acid biosynthesis | 110025 | 61738 |
